# Nitrotyrosine Density of Rabbit Urinary Bladder Muscle and Mucosa Measured via Western Blotting and 96-Well Plate Analysis

**DOI:** 10.5402/2012/618247

**Published:** 2012-02-29

**Authors:** Brittany Fitzpatrick, Catherine Schuler, Robert E. Leggett, Robert M. Levin

**Affiliations:** ^1^Department of Pharmaceutical Sciences, Albany College of Pharmacy and Health Sciences, 106 New Scotland Ave, Albany, NY 12208, USA; ^2^Department of Research, Stratton VA Medical Center, 113 Holland Ave, Albany, NY 12208, USA; ^3^Division of Urology, Albany Medical College, 25 Hackett Blvd, Albany, NY 12208, USA

## Abstract

*Purpose*. Nitrotyrosine was quantitated in rabbit bladder muscle and mucosa using two analytical systems: Western blotting analyses and a 96-well plate quantitative analysis kit. *Materials and Methods*. Rabbit bladder muscle and mucosa were obtained from control rabbits. For the Western analysis, the samples were loaded into a SDS page gel and then transferred to a PVDF membrane. The optical density was measured using a Kodak Scanner. Using the 96-well plate, the samples and standards were loaded, incubated with primary and secondary antibody, washed and vacuumed with 10x wash buffer three times between each incubation period. Stop buffer was added to the plate and the results were quantified via the plate reader. *Results*. For both muscle and mucosa tissue, the optical density readings were linear with tissue concentration; the concentration of nitrotyrosine in the mucosa was significantly higher than in the muscle. However, whereas the Western blot analysis is based on relative optical densities, the 96-well plate kit provides a truly quantitative analysis. *Discussion*. Mucosa tissue displayed a higher density of nitrotyrosine than did detrusor muscle tissue. This may well be due to the significantly higher metabolic activity of the mucosa compared to the muscle.

## 1. Introduction

Urinary bladder problems are a common health problem amongst both men and women. Finding the underlying cause of bladder dysfunction is important in determining appropriate treatment regimens. 

The bladder is integrated with both parasympathetic and sympathetic nerves. Stimulation of sympathetic nerves results in filling of the bladder via alpha and beta receptors. The parasympathetic nerves stimulate the emptying of the bladder via muscarinic receptors [1, 2].

Benign prostatic hyperplasia (BPH) most commonly results in bladder dysfunction in aging men and the symptoms include urgency, frequency, and nocturia [1–3]. Obstructive symptoms of this disease state include reduced flow rate, reduced micturition pressure, overactive bladder syndrome, and incomplete emptying. Although the symptoms are related to the enlarged prostate, the medical problems are related to compression of the urethra and the creation of a partial outlet obstruction [3, 4]. The functional problems and changes that are seen in the human bladder can be mirrored in a rabbit model of partial outlet obstruction [5, 6].

 Partial outlet obstruction results in compensation which eventually progresses into decompensation. In compensation the bladder mass increases, there is hypertrophy in the bladder smooth muscle, mucosal hyperplasia, and angiogensis. Even in the presence of these structural changes, there is normal bladder function. In decompensation, there is a further increase in bladder mass, angiogenesis, and along with this there is a decrease in bladder compliance and contractile function. Interestingly, even though there is angiogenesis in both the compensated and decompensated bladders, the increased vasculature is found around the hypertrophied muscle and not through the muscle bundles [4, 7, 8].

 The shift from compensation to decompensation is directly linked to ischemia/reperfusion which accompany hypoxia of the hypertrophied bladder. The ischemia/reperfusion directly results in an increase in free radicals and is the cause of the oxidative stress experienced by the tissue. We have identified four indicators that are related to the shift from compensation to decompensation. The four markers that have been identified include reduced blood flow and presence of hypoxia; reduced cholinergic nerve density (denervation); reduced mitochondrial function and metabolic energy production; decreased sarcoplasmic reticulum function resulting in calcium dysregulation of intracellular resulting in increased free intracellular calcium. These four markers have also been noted in men with BPH. Partial outlet obstruction is, therefore, directly linked to the observed increase in protein oxidation and nitration [4, 6, 9, 10]. Nitrotyrosine is the product of reactive nitrogen species of free radicals and has generally been semiquantitated by Western blot analyses using relative optical densities of control tissue compared with obstructed tissue [11–13].

 In the current study, we compared the results of Western blot analyses with the quantitative results of a nitrotyrosine analytical kit using 96-well plate technology.

## 2. Methods

All use of rabbits in this study was approved by the IACUC of the Stratton VA Medical Center.

### 2.1. Tissues

 Control bladders from New Zealand white rabbits were utilized for these experiments (*N* = 4). The bladder smooth muscle and mucosa were separated by blunt dissection, frozen under liquid nitrogen, and stored at −80°C until analyzed. At the time of analysis, the tissue samples were homogenized in 50 mM Tris buffer (pH = 8.0) using a Polytron homogenizer, centrifuged at 2500 RPM's for 10 minutes and the pellet discarded. The analysis utilized the supernates at concentrations of 100, 50, and 25 mgs/mL.

### 2.2. Nitrotyrosine: Western Blot Procedure

Two gels were prepared and the standards and tissue samples were loaded. The gels were placed in a Bio-Rad unit and run at 150 volts for 60 minutes to allow the proteins to separate. After this, they were removed from the unit and put in transfer buffer along with 2 PVDF membranes which were previously washed in methanol for 10 seconds. The membranes and gels were placed in cassettes, then put in a unit filled with transfer buffer, and run overnight at 22 volts. This allowed the standards and samples to be transferred from the gels to the PVDF membranes. The PVDF membranes were removed from the gels and placed in blotto for 30 minutes at 37°C. After this the membranes were incubated in primary and then the secondary antibody for 30 and 45 minutes, respectively at 37°C. They were washed and rocked in TTBS at room temperature 5 separate times for 5 minutes each between the primary and secondary antibody applications and also after the secondary antibody. The membranes were covered with 1 mL of ECL Plus for 2 minutes and then placed in a sealed bag. They were scanned via the Kodak Image Station, and the pictures were then analyzed. 

### 2.3. 96-Well Plate Quantitation

The standards, muscle, and mucosa tissue samples were loaded into the 96-well plate. Primary and secondary antibodies were loaded and incubated in the well plates with the sample. Following each incubation with the primary and secondary antibodies, the well plates were vacuumed and washed with 10x wash buffer for 3 times. After the secondary antibody incubation, stop solution was added to the well plate, and it was analyzed via the plate reader.

## 3. Results

**Figure 1:**
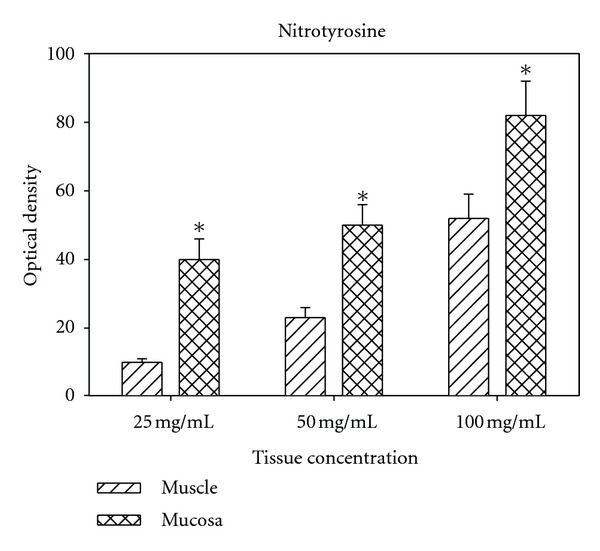
Shows the values of optical density via nitrotyrosine: Western blotting analysis. *: significantly different from muscle.

Figure 1 displays the results of the Western blot analyses using arbitrary optical density units. As can be observed, the optical density increases in proportion to the tissue concentration, and at each tissue concentration the mucosa showed a significantly higher concentration of nitrotyrosine than the bladder smooth muscle. 

**Figure 2:**
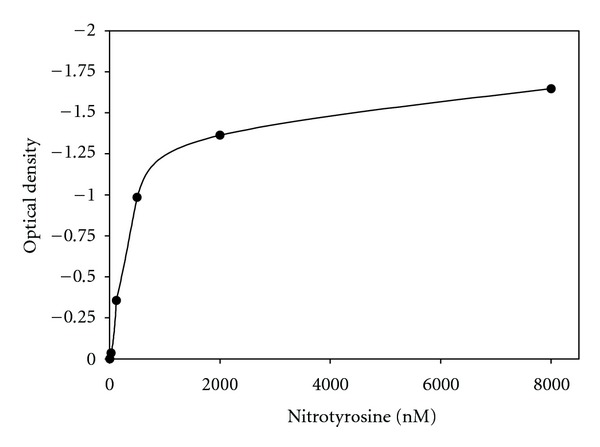
Shows the values of optical density via 96-well plate analysis in linear terms.

**Figure 3:**
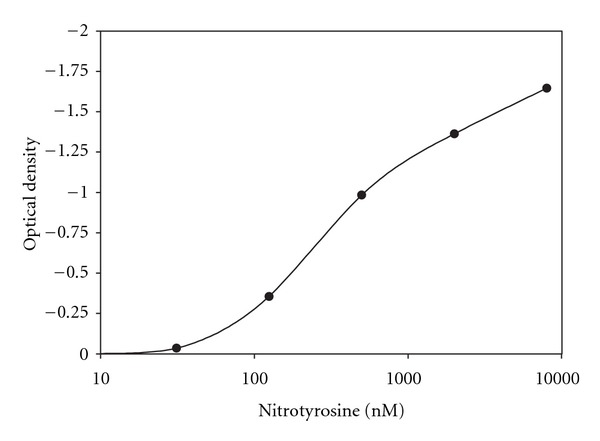
Shows the values of optical density via 96-well plate analysis in logarithmic terms.

**Figure 4:**
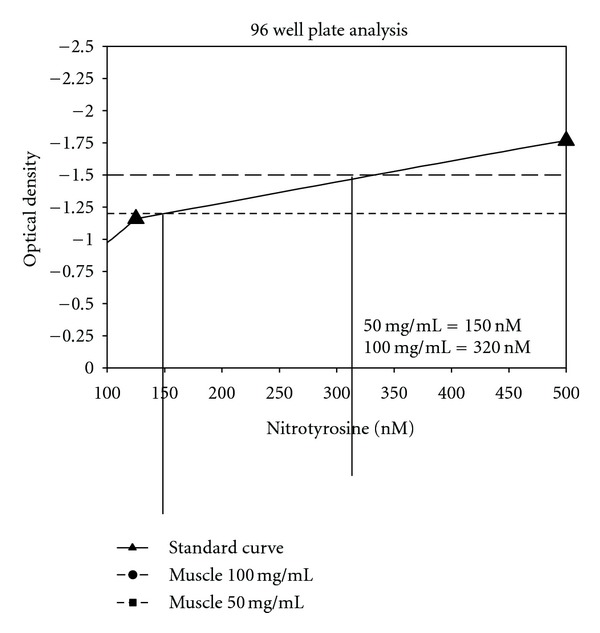
Shows the values of optical density via 96-well plate analysis of bladder smooth muscle at a tissue concentration of 50 and 100 mg/mL.

**Figure 5:**
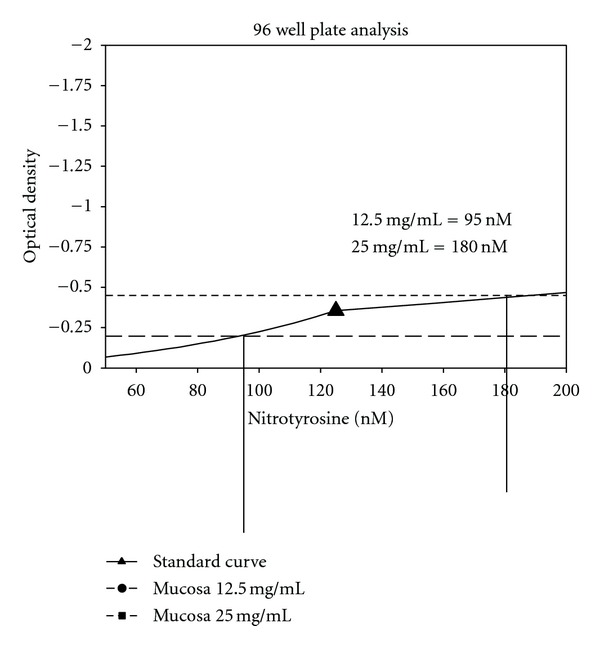
Shows similar curves for bladder mucosa.

**Figure 6:**
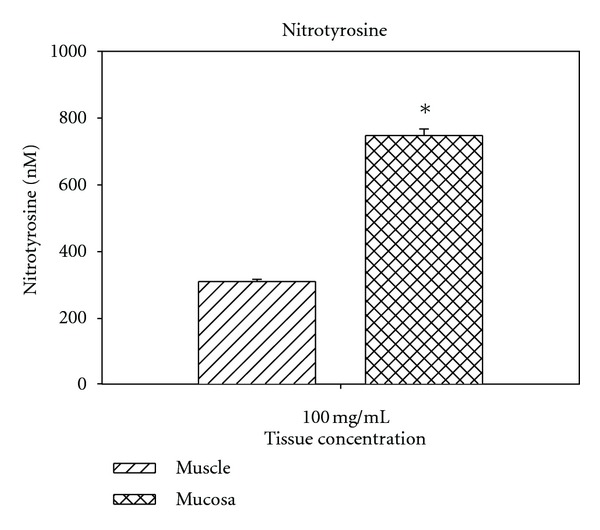
Shows the concentration of nitrotyrosine for muscle and mucosa normalized to the original 100 mg/mL tissue concentration. *: significantly different from muscle.

 The 96-well plate analysis conveys more quantitative results.  Figure 2 displays the linear dose response curve to the standards provided in the kit. Unlike other standard curves, this one goes from 0 to negative 1.5. However, the range is from 500 to 8000 nM nitrotyrosine.  Figure 3 shows the standard curve in logarithmic terms.  Figure 4 shows the results for bladder smooth muscle measured at 50 and 100 mg/mL. The curve shows the optical density readings for the two concentrations and their intersection points on the standard curve and gives the quantitative nitrotyrosine data for the two concentrations.  Figure 5 shows similar curves for bladder mucosa.  Figure 6 shows the concentration of nitrotyrosine for muscle and mucosa normalized to the original 100 mg/mL tissue concentration. Similar to the Western blot analyses, the mucosa had a significantly greater concentration of nitrotyrosine than that of the muscle.

## 4. Discussion

Nitrotyrosine density increases as a result of ischemic damage [14, 15]. Ischemic damage causes the mitochondrial damage and the sarcoplasmic reticulum to release calcium from their binding sites [16–21]. This leads to an increase in cytosolic calcium levels and ultimately an increase in oxidative stress. These free radicals result in damage to superoxide dismutase (SOD) and catalase enzymes. SOD and catalase work together within the same pathway to neutralize free radicals. By decreasing their concentrations, there is a decrease in their ability to neutralize these radicals thus increasing the damaging effect of the free radicals.

Nitrotyrosine is a common marker for RNS free-radical damage [13, 22]. Optical density of the Western blot techniques is a semiquantitative measure of the stress that the bladder tissue is under and is based on the relative optical density of a control tissue compared to the pathological tissue. The current study was aimed at first to show that there was a concentration-dependent change in optical densities of increasing concentrations of tissues using the Western blot analyses and to determine the relative density of nitrotyrosine in bladder control smooth muscle and mucosa. We then compared this with the data derived from a quantitative ELISA methodology using a 96-well plate system. 

 When comparing muscle and mucosa tissue, the nitrotyrosine concentration in the mucosal tissue was statistically significantly higher than the muscle tissue. This indicates that there is more oxidative damage within the control mucosal tissue than in the muscle tissue. This may be due to the fact that the mucosa has significantly higher metabolic activity than the muscle and has a significantly lower high-energy phosphate concentration [23–25].

 In conclusion, both the Western blot analyses and quantitative 96-well plate kit analyses showed linearity with increasing concentrations of tissue, and that the mucosal concentration of nitrotyrosine was significantly greater than the concentration in the muscle. The advantage of the 96-well plate technology is that it is a quantitative assay rather than a relative qualitative assay.
